# Transcriptomic response of *Saccharomyces cerevisiae* to octanoic acid production

**DOI:** 10.1093/femsyr/foab011

**Published:** 2021-02-18

**Authors:** Leonie Baumann, Tyler Doughty, Verena Siewers, Jens Nielsen, Eckhard Boles, Mislav Oreb

**Affiliations:** Faculty of Biological Sciences, Institute of Molecular Biosciences, Goethe University Frankfurt, Max-von-Laue Straße 9, 60438 Frankfurt am Main, Germany; Department of Biology and Biological Engineering, Chalmers University of Technology, Kemivägen 10, SE-41296 Gothenburg, Sweden; Department of Biology and Biological Engineering, Chalmers University of Technology, Kemivägen 10, SE-41296 Gothenburg, Sweden; Novo Nordisk Foundation Center for Biosustainability, Chalmers University of Technology, Kemivägen 10, SE-41296 Gothenburg, Sweden; Department of Biology and Biological Engineering, Chalmers University of Technology, Kemivägen 10, SE-41296 Gothenburg, Sweden; Novo Nordisk Foundation Center for Biosustainability, Chalmers University of Technology, Kemivägen 10, SE-41296 Gothenburg, Sweden; BioInnovation Institute, Ole Maaløes Vej 3, DK-2200 Copenhagen N, Denmark; Faculty of Biological Sciences, Institute of Molecular Biosciences, Goethe University Frankfurt, Max-von-Laue Straße 9, 60438 Frankfurt am Main, Germany; Faculty of Biological Sciences, Institute of Molecular Biosciences, Goethe University Frankfurt, Max-von-Laue Straße 9, 60438 Frankfurt am Main, Germany

**Keywords:** *octanoic acid*, *transcriptome response to medium-chain fatty acids*, *RNA-Seq*, *RPL40B*

## Abstract

The medium-chain fatty acid octanoic acid is an important platform compound widely used in industry. The microbial production from sugars in *Saccharomyces cerevisiae* is a promising alternative to current non-sustainable production methods, however, titers need to be further increased. To achieve this, it is essential to have in-depth knowledge about the cell physiology during octanoic acid production. To this end, we collected the first RNA-Seq data of an octanoic acid producer strain at three time points during fermentation. The strain produced higher levels of octanoic acid and increased levels of fatty acids of other chain lengths (C6–C18) but showed decreased growth compared to the reference. Furthermore, we show that the here analyzed transcriptomic response to internally produced octanoic acid is notably distinct from a wild type's response to externally supplied octanoic acid as reported in previous publications. By comparing the transcriptomic response of different sampling times, we identified several genes that we subsequently overexpressed and knocked out, respectively. Hereby we identified *RPL40B*, to date unknown to play a role in fatty acid biosynthesis or medium-chain fatty acid tolerance. Overexpression of *RPL40B* led to an increase in octanoic acid titers by 40%.

## Teaser

RNA-Seq analysis of a *Saccharomyces cerevisiae* strain producing octanoic acid identified *RPL40B* as an important gene for improved production of medium-chain fatty acids.

## INTRODUCTION

Medium-chain fatty acids like octanoic acid (C8 fatty acid) have a wide range of applications in antimicrobials, surfactants and cosmetics, and can also serve as precursors for biofuels (Huang *et al*. 2011; Rosenblatt *et al*. 2017; Sarria, Kruyer and Peralta-Yahya 2017; Henritzi *et al*. 2018). At present, octanoic acid and its derivatives are produced from oil seed crops or crude oil (Yan and Pfleger 2020). In comparison to petrochemical industry, the extraction from oily plants, such as oil palm, might appear environmentally less harmful but there are rising concerns about its extensive cultivation. Oil palm cultivation is often associated with deforestation of rainforest and the ecological consequences thereof, and in addition competes with food production (Schmidt 2015). The engineering of microbes for octanoic acid production from renewable biomass is therefore a promising alternative (Hu *et al*. 2019; Yan and Pfleger 2020).

Yeast produces fatty acids in the fatty acid synthesis cycle through action of the cytosolic fatty acid synthase (FAS), a large enzymatic complex encoded by the two genes *FAS1* and *FAS2*. Naturally, *Saccharomyces cerevisiae* produces mostly saturated and monounsaturated long-chain fatty acids with chain lengths of C16 and C18 (Klug and Daum 2014). There are different ways to redirect production from long-chain to short- and medium-chain fatty acids. Here, we use a yeast strain that carries a modified variant of *FAS1*, namely *FAS^RK^* (Gajewski *et al*. 2017). This enzyme contains an amino acid exchange from arginine to lysine (‘R1834K’) in the malonyl-palmitoyl transferase (MPT) domain of Fas1p. Thereby, the loading of the precursor malonyl-CoA is reduced, leading to premature release of short acyl chains, such as octanoyl-CoA. Octanoyl-CoA is then hydrolysed by thioesterases and free octanoic acid is released (Gajewski *et al*. 2017). For an economically feasible production, further strain improvements and process optimizations are necessary (Baumann *et al*. 2020).

To increase titers, it is essential to advance the understanding of the producer strain's physiology in the course of octanoic acid production (Jarboe, Liu and Royce 2011). To our knowledge, such an analysis has not been undertaken yet. So far, two transcriptome-wide studies have been performed with extracellular addition of octanoic acid to wild type strains (Legras *et al*. 2010; Liu *et al*. 2013). In the first one, a microarray analysis showed that incubation with 0.05 mM (7 mg/L) octanoic acid for 20 min caused oxidative stress and a similar response to decanoic acid was observed. Overall, 75 genes were found to be differentially regulated in comparison to the non-exposed control. Pdr12 was identified as the main involved transporter and the transporter Tpo1 was shown to play a minor role in octanoic acid expulsion (Legras *et al*. 2010). In the second study, the exposure to 0.3 mM (43 mg/L) octanoic acid until mid-log growth phase revealed differential expression of at least 2-fold of 136 genes in comparison to the non-exposed control. Here, membrane leakiness was identified as a possible mechanism of cell disruption and increasing the oleic acid content was shown to enhance yeast tolerance to octanoic acid (Liu *et al*. 2013).

To get a comprehensive insight into expression changes during production of octanoic acid, we performed RNA-Seq in three different growth phases of an *S. cerevisiae* octanoic acid producer strain and a reference strain. We used expression profiles from the different growth phases to guide strain engineering efforts and achieved a 40% increase in octanoic acid production.

## RESULTS

### Fermentation profiles of an octanoic acid producer compared to reference

To get the first insight into transcriptional changes in different growth phases associated with octanoic acid production, we performed RNA-Seq transcriptome profiling. As a background strain we used SHY34, (Wernig *et al*. 2020) a triple knockout strain (∆*fas1*∆*fas2*∆*faa2*). In this strain the two *FAS* genes are knocked out as well as *FAA2*, encoding a fatty acyl-CoA synthetase, deletion of which prevents degradation of short- and medium-chain fatty acids (Leber *et al*. 2016; Henritzi *et al*. 2018). We transformed this strain either with a reference plasmid containing *FAS1* and *FAS2* in their wild type form fused into one sequence, i. e. *fusFAS^wt^*, or with a plasmid carrying *FAS1^RK^* and *FAS2* fused into one sequence, namely *fusFAS^RK^*. The *fusFAS^RK^* sequence contains an amino acid exchange from arginine to lysine in the MPT domain (R1834K), generating an octanoic acid producer (Wernig *et al*. 2020).

**Figure 1.**
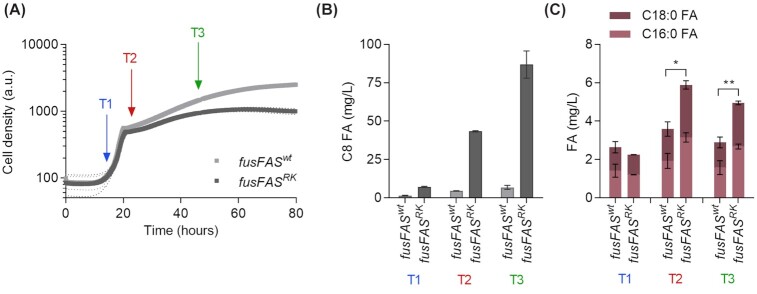
Characterization of growth and octanoic acid production. **(A)** Growth was monitored on a cell growth quantifier in triplicates for strains SHY34 + *fusFAS^wt^* (reference strain) and SHY34 + *fusFAS^RK^* (octanoic acid producer). Sampling times are indicated with arrows (T1 = 14 h; T2 = 22 h and T3 = 46 h). **(B, C)** C8, C16:0 and C18:0 fatty acids (FA) were extracted and quantified via GC. *n *= 3, error bars = ± standard deviation. Statistical analysis was performed using two-tailed t test (*P < 0.05, **P < 0.01).

The two strains grew with a similar growth rate in exponential phase and only after the diauxic shift, growth was slower in the octanoic acid producer strain compared to the reference strain leading to a lower final OD in the octanoic acid producer (Fig. 1A). Such impairment of growth has similarly been observed for non-producers spiked with octanoic acid (Henritzi *et al*. 2018). Samples were taken in early exponential growth phase (14 h, T1), shortly after the diauxic shift (22 h, T2) and in ethanol consumption phase (46 h, T3), respectively. To confirm octanoic acid production, samples were taken from the culture supernatants at each sampling time for fatty acid extraction and quantification via GC measurement (Fig. 1B). In the reference strain, octanoic acid was produced in negligible amounts (< 7 mg/L) at all three sampling times whereas in the octanoic acid producer, the amount increased gradually from 7 mg/L (T1) to 43 mg/L (T2) to 87 mg/L (T3). We also observed an enhanced production of other short- and medium-chain fatty acids, namely C6, C10 and C12 fatty acids, in the octanoic acid producer strain, with the most pronounced difference to the reference strain at T3 (Figure S1, Supporting Information). Interestingly, even secreted amounts of saturated long-chain fatty acids, i.e. C16:0 and C18:0 fatty acids, were higher in the octanoic acid producer compared to the reference strain at T2 and T3 (Fig. 1C).

### Analysis of transcriptomic response in different phases of octanoic acid production

From the samples described above, we extracted RNA and performed RNA-Seq analysis. A principal component analysis (PCA) of the RNA-Seq data set revealed a close clustering of the three replicates at each sampling time in both strains (Figure S2, Supporting Information). The data of the reference strain and the octanoic acid producer strain also clustered closely together, suggesting a limited number of differentially expressed genes (Figure S3, Supporting Information). At T1, only 15 genes were upregulated (log_2_FC > 1, FDR < 0.01) in the octanoic acid producer in comparison to the reference strain—at T2 this was the case for 29 genes and at T3 for 85 genes. The number of downregulated genes was in a similar range, with 11 genes downregulated at T1, 44 genes at T2 and 144 genes at T3. The number of differentially expressed genes between the two strains therefore increased with fermentation time. A list of all differentially expressed genes can be found in Supplementary File 1. GO term enrichment analyses for all three sampling times are attached as Supplementary File 2.

**Figure 2.**
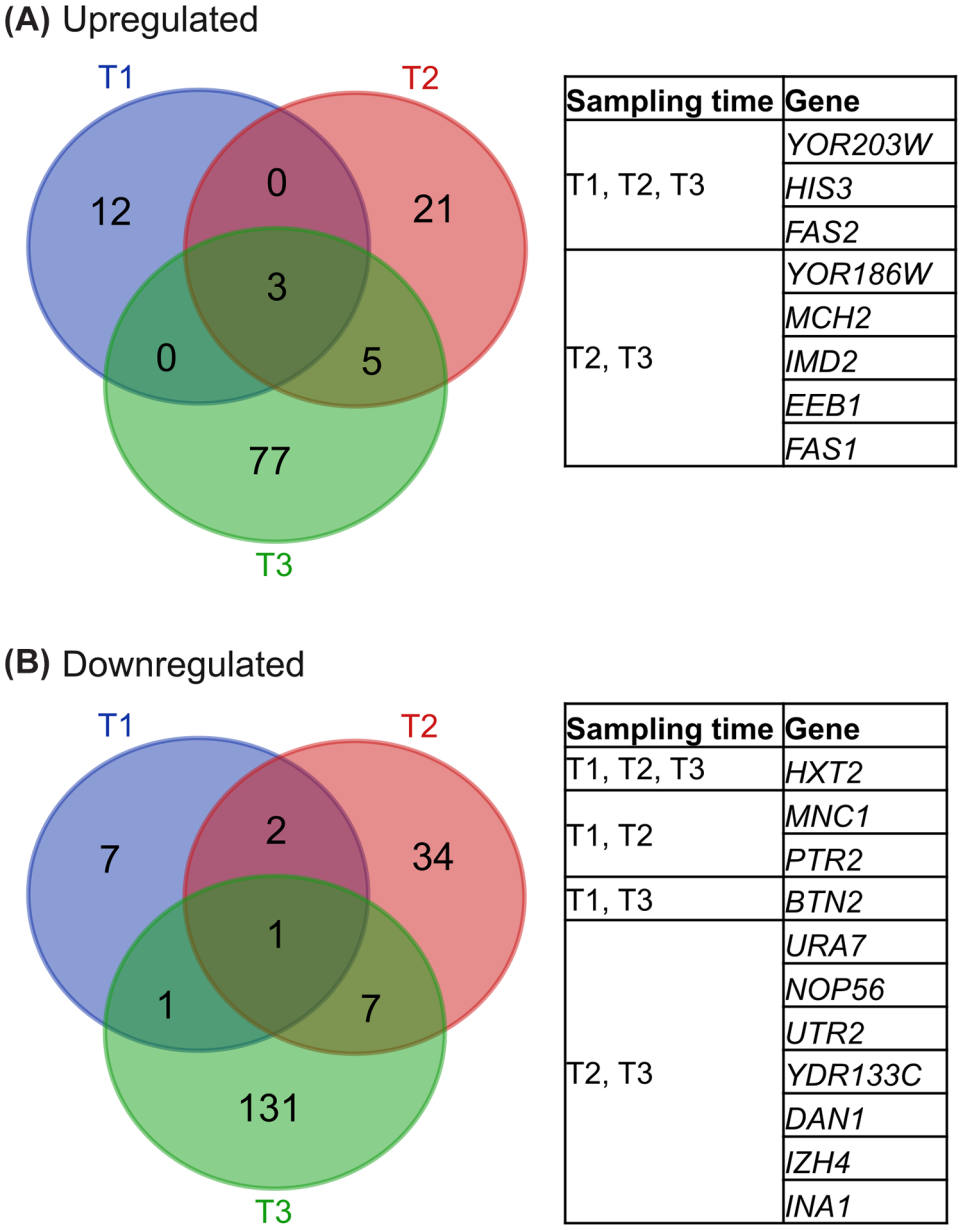
Differential gene expression for the octanoic acid producer compared to reference. Number of genes that are upregulated **(A)** or downregulated **(B)** in the octanoic acid producer strain in comparison to the reference strain (log_2_FC > 1, FDR < 0.01) between sampling times (T1 = 14 h; T2 = 22 h and T3 = 46 h). Gene lists include genes that are differentially regulated at more than one sampling time as indicated.

To get a comprehensive view of the transcriptomic landscape of the octanoic acid producer in different growth phases, we compared the RNA-Seq data of the three sampling times (Fig. 2). We observed only eight genes upregulated and 11 genes downregulated at more than one sampling time compared to the reference. For upregulation, these included *FAS1^RK^*, *FAS2* as well as *HIS3*–the latter being the selection marker of the *fusFAS^RK^* plasmid used in the experiment. A higher copy number or expression of *fusFAS^RK^* was recently shown to increase octanoic acid titers (Wernig *et al*. 2020). It is likely that the expression was upregulated in the octanoic acid producer to ensure that enough long-chain fatty acids are produced for cell growth since chain length control in *fusFAS^RK^* is leaky (Gajewski *et al*. 2017). *YOR203W* was found to be upregulated at all sampling times, however, according to the SGD database, it is unlikely to encode a functional protein, and its assigned sequence partially overlaps with *HIS3*, suggesting an incorrect assignment in the RNA-Seq read mapping. *HXT2*, a glucose transporter, was the only gene found to be downregulated at all three sampling times.

### Comparison to published transcriptome data

A total of two other transcriptome-wide studies have been performed so far, however, both of them with extracellular addition of octanoic acid to wild type *S. cerevisiae* strains (Legras *et al*. 2010; Liu *et al*. 2013). In the first study, a microarray analysis by Legras and colleagues, (Legras *et al*. 2010) 75 genes were found to be differentially regulated (log_2_FC ≥ 0.3, FDR < 0.05) in comparison to the non-exposed control after incubation with only 0.05 mM (7 mg/L) octanoic acid for the short period of 20 min (Legras *et al*. 2010). In the second study by Liu *et al*. (2013) the exposure to six times the amount, i. e. 0.3 mM (43 mg/L) octanoic acid, led to differential expression (log_2_FC > 1, *P* < 0.01) in 136 genes in comparison to the non-exposed control (Liu *et al*. 2013). Interestingly, the overlap between these two published studies and our analysis was limited to a very small number of genes (Figures S4 and S5, Supporting Information). In all three studies the transporter Pdr12 was found to be upregulated (RNA-Seq: T3), which has previously been associated with octanoic acid expulsion (Legras *et al*. 2010). However, additional plasmid-based expression of Pdr12 has not led to an increase in octanoic acid titers in our hands, suggesting that endogenous expression is sufficient (Figure S6, Supporting Information).

To understand expression changes in the octanoic acid producer, we wanted to further analyse the effect of differentially expressed genes. To limit the number of genes for further analysis, we decided to focus on those genes that were differentially expressed at more than one sampling time in our data. We reasoned that such genes might have a more profound effect on octanoic acid production and constitute more promising targets for engineering than genes that are differentially regulated at only one sampling time.

### Overexpression of identified genes with a multi-copy plasmid library

**Figure 3.**
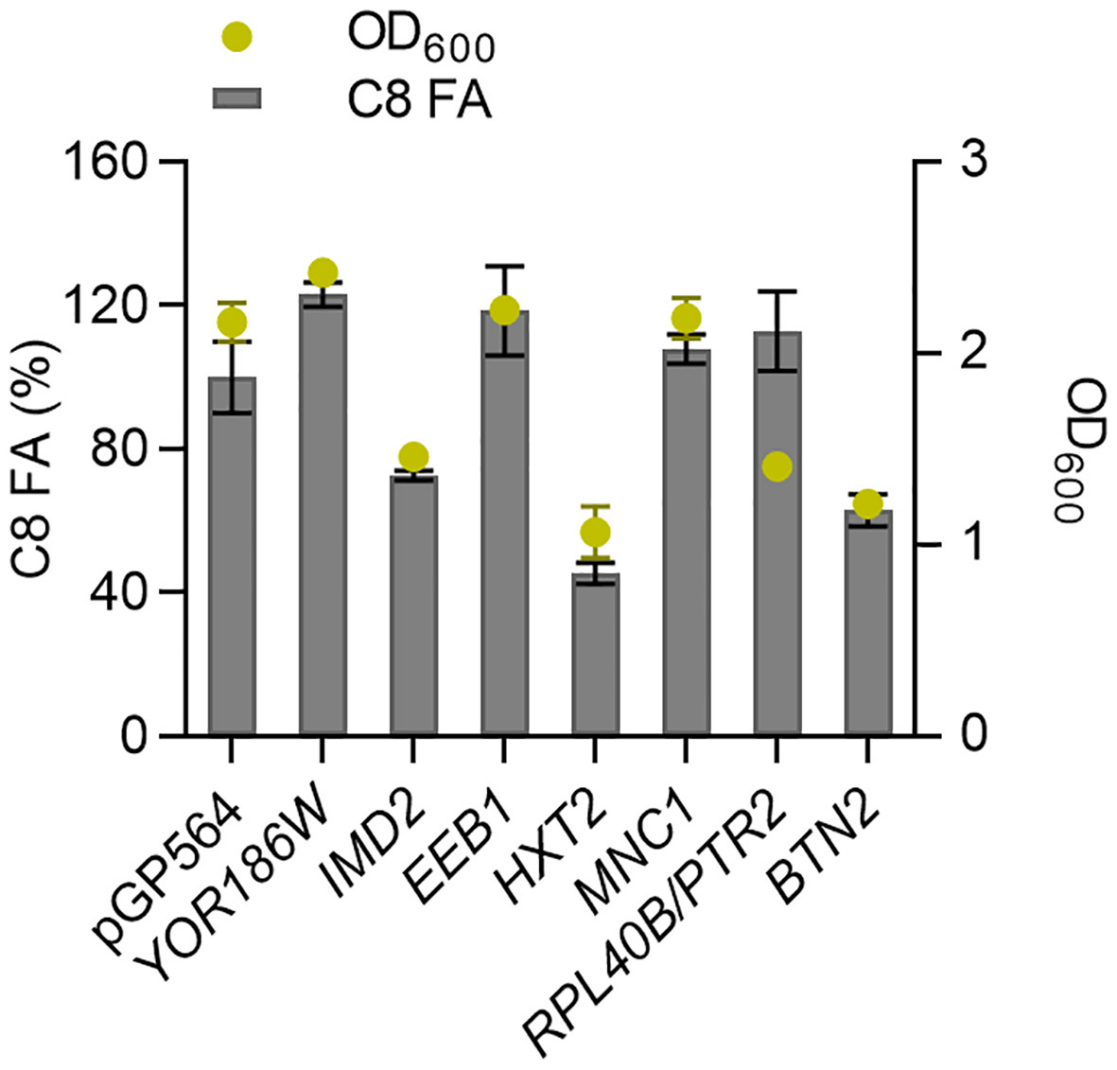
Fermentation of octanoic acid producer with overexpressed genes. The octanoic acid producer strain LBY38 was transformed with plasmids of a multi-copy library or a control plasmid (pGP564) and cultured for 18 h in buffered SCD^−leu^ medium. Tested library plasmids contained the genes indicated on the *x*-axis. Octanoic acid was extracted and quantified by GC measurement, and is shown in % of the control (pGP564). *n* = 2, error bars = ± standard deviation.

We decided to test the effect of plasmid-based overexpression of all genes that were found to be up- or down-regulated, respectively, in at least two sampling times in our data (see gene tables in Fig. 2). In addition, we also tested the effect of overexpression of some genes overlapping with data from Liu and colleagues (Liu *et al*. 2013). For overexpressing the respective genes, we used a plasmid-library (Jones *et al*. 2008) that contains the majority of the yeast genome cloned as approximately 10 kb fragments into multi-copy plasmid backbones. Two genes (*DAN1*, *NOP56*) were not represented in the library and were therefore cloned separately on a multi-copy plasmid backbone. To avoid plasmid burden effects, we tested the plasmids in a strain that contains *FAS1^RK^* and *FAS2* in their wild type loci (LBY38). Towards the end of the exponential growth phase (18 h) we evaluated the octanoic acid titers for some of the strains and observed a variation of titers in accordance with differing growth (Fig. 3). LBY38 containing plasmids with *IMD2*, *HXT2* or *BTN2*, respectively, had OD_600_ values and titers of only about 50% of the control, indicating delayed growth and therefore lower octanoic acid production.

**Figure 4.**
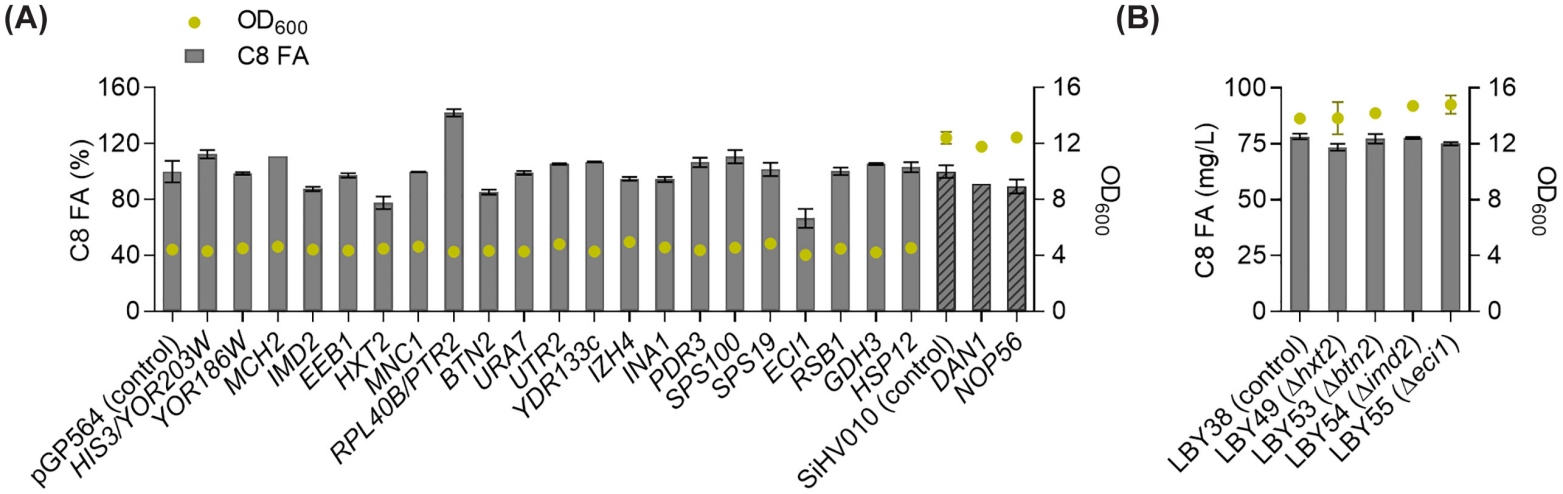
Test of overexpression and deletion of targets in octanoic acid producer. **(A)** The octanoic acid producer strain LBY38 was transformed with plasmids of a multi-copy library and cultured in buffered SCD^−leu^ medium. Tested library plasmids contained the genes indicated on the *x*-axis (dark grey bars). The genes *DAN1* and *NOP56* were not part of the library and therefore were cloned individually into SiHV010 multi-copy backbone, transformed into LBY38 and cultured in buffered YPD^hyg^ (striped bars). Titers are shown in % of the respective control strains. **(B)** A total of four genes were knocked out individually in LBY38 and the strains were fermented in buffered YPD. For all samples, octanoic acid was extracted after 46 h of growth and quantified by GC measurement. *n* = 2, error bars = ± standard deviation.

In ethanol consumption phase (46 h), all strains had reached similar OD_600_ values, which facilitated comparison between octanoic acid titers (Fig. 4A). After 46 h of fermentation, the expression of the library plasmid containing two neighboring genes of interest, *RPL40B* and *PTR2*, yielded a 40% higher octanoic acid titer; whereas *HXT2*, *BTN2* and *IMD2* expression led to a 25%, 15% and 13% decrease, respectively. The expression of the library plasmid containing *ECI1* led to a decrease in titer to about 60% of the reference. All other plasmids did not (< 10% difference) influence octanoic acid titers. To further characterize the effects on octanoic acid production, we generated individual knockout strains for *HXT2*, *BTN2*, *IMD2* and *ECI1* in the LBY38 background. We analysed octanoic acid titers after 46 h of growth in buffered SCD medium. All four knockout strains, ∆*hxt2*, ∆*btn2*, ∆*imd2* and ∆*eci1*, showed similar growth as well as octanoic acid titers as the control LBY38 (Fig. 4B).

### Overexpression of *RPL40B* leads to increase in octanoic acid production

**Figure 5.**
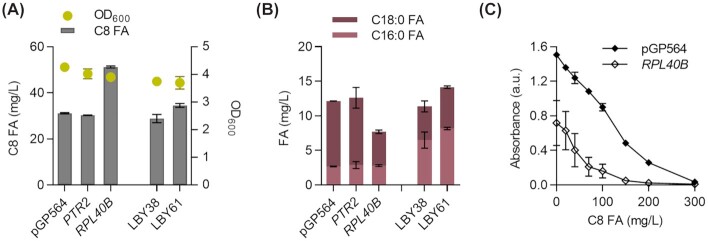
Effect of *PTR2* and *RPL40B* overexpression. **(A, B)** The octanoic acid producer strain LBY38 was transformed with plasmids containing *PTR2*, *RPL40B* or the control vector (pGP564). A second copy of *RPL40B* was integrated into the genome under control of *pPGK1* in the *PYK2* locus in LBY38 resulting in strain LBY61. Strains were cultured in buffered SCD medium and fatty acids (FA) were extracted after 46 h and quantified by GC measurement. *n* = 2, error bars = ± standard deviation. **(C)** Non-producer CEN.PK2–1C containing either the vector pGP564 (control) or *RPL40B* were inoculated in triplicates to OD_600 _= 0.05 and supplied with 0–300 mg/L octanoic acid. Growth was measured by absorbance after 20 h. *n* = 3, error bars = ± standard deviation.

To determine which of the two genes - *RPL40B* or *PTR2* – is responsible for the observed positive effect, we cloned each with its native promotor and terminator on a separate plasmid backbone. The fermentation showed that the plasmid-based expression of *RPL40B* caused an increase in octanoic acid titers of 40%, whereas *PTR2* expression did not influence octanoic acid titers (Fig. 5A). The amount of secreted saturated C18 fatty acid was lower in the strain with plasmid-based *RPL40B* expression compared to the reference (Fig. 5B). When introducing a second copy of *RPL40B* into the genome under the control of the strong *pPGK1* promoter, we could not observe this increase in titers (Fig. 5A), suggesting that the native *RPL40B* regulatory elements are required for the positive effect on octanoic acid production. To investigate the effect of *RPL40B* on robustness towards octanoic acid, we transformed the non-producer CEN.PK2–1C with either a control plasmid or the multi-copy plasmid containing *RPL40B*. After incubation for 20 h in a 96-well plate (without shaking) with 0–300 mg/L octanoic acid, the strain with plasmid-based expression of *RPL40B* showed strongly decreased growth under all octanoic acid concentrations and even without addition of octanoic acid (Fig. 5C).

## DISCUSSION

To date, no transcriptomics data of an *S. cerevisiae* octanoic acid producer strain was available. To fill this gap, we generated a high-quality RNA-Seq data set for three sampling times during fermentation. By comparing the growth, fatty acid and expression profile of the producer to a reference strain, we were able to get a profound insight into transcriptomic changes during octanoic acid production.

In the course of fermentation, octanoic acid biosynthesis increased in the producer strain from 7 mg/L after 14 h to 87 mg/L after 46 h. This increase correlated with the increase in differential expression in comparison to the reference strain, suggesting that increasing octanoic acid titers influence overall strain physiology. In terms of growth, the two strains behaved similarly until the diauxic shift when growth of the octanoic acid producer slowed down more quickly and reached lower final OD values. The main production of octanoic acid started shortly before or during the diauxic shift, which is consistent with the production profile observed in our previous studies (Gajewski *et al*. 2017; Wernig *et al*. 2020). Although we did not measure the concentrations of glucose and ethanol in the course of fermentations shown in Fig.   1, the sampling points can be clearly assigned to the glucose (T1) and ethanol consumption phases (T2 and T3), respectively, based on the previously published correlation of precisely measured growth curves and carbon source profiles (Bruder *et al*. 2016). This implies that octanoic acid production mainly occurs during the consumption of ethanol, presumably due to an increased supply of the precursor molecule acetyl-CoA on this carbon source. The onset of octanoic acid production parallels the slowdown in growth; this can probably be attributed to toxic effects of octanoic acid on the cell physiology (Viegas *et al*. 1989; Legras *et al*. 2010; Liu *et al*. 2013; Henritzi *et al*. 2018), which is further potentiated in the presence of ethanol (Viegas, Sá-Correia and Novais 1985; Viegas *et al*. 1989). Octanoic acid disrupts the composition of the yeast plasma membrane leading to increased leakage and at high concentrations cell death (Legras *et al*. 2010; Liu *et al*. 2013). This effect was shown to be mitigated by an increase in the average chain length of membrane fatty acids, i.e. more C18 than C16 fatty acids, as well as an increase in unsaturated fatty acid, i.e. oleic acid (C18:1; Liu *et al*. 2013; Besada-Lombana *et al*. 2017). In the culture supernatant, unsaturated long-chain fatty acids were not detectable. Interestingly, at T2 and T3, we observed that the octanoic acid producer secreted more saturated long-chain fatty acids of chain lengths C16 and C18 than the reference strain. Since the strain used in the experiment is a ∆*fas1*∆*fas2*∆*faa2* deletion strain, its only possibility to produce long-chain fatty acids is the leaky synthesis through plasmid-encoded *fusFAS^RK^*. Even though the ‘R1834K’ amino acid exchange in the MPT domain of *FAS1* leads to premature release of short- and medium-chain fatty acids from the FAS cycle, this regulation is not completely tight (Gajewski *et al*. 2017). As the pressure to synthesize enough long-chain fatty acids, which are essential for growth and survival, is probably high, the copy number of the plasmid containing *fusFAS^RK^* seems to have been increased. The evidence for this is twofold: Firstly, we saw higher expression of the *fusFAS^RK^* construct with RNA-Seq for all time points in the octanoic acid producer strain and secondly, the expression of the marker gene on the plasmid, *HIS3*, was increased accordingly. In summary, this suggests that gene expression is not optimized for a maximum production of octanoic acid but rather ideal for producing sufficient long-chain fatty acids to survive and mitigate toxic effects of octanoic acid. This could also explain the limit in octanoic acid production that we seem to encounter, as the precursor malonyl-CoA is increasingly used for C16 and C18 rather than for C8 fatty acid biosynthesis.

To get a deeper understanding of transcriptional changes, we analyzed differential expression at the three sampling times in the producer compared to the reference. For this comparison, we chose a log_2_FC of at least 1 as a threshold (FDR < 0.01) and chose a subset of the hits for further analysis. We evaluated the effect of additional, plasmid-based expression of all genes that were found to be up- or downregulated, respectively, at more than one RNA-Seq sampling time in the octanoic acid producer. For this purpose, we chose to use a multi-copy library which contains the entire yeast genome cloned onto 2µ backbone plasmids and each plasmid contains roughly 2–10 genes (Jones *et al*. 2008). Even though the other genes on the plasmid might also affect octanoic acid production, it is unlikely that they so much influence titers as to mask the possible effect of the gene-of-interest. The expression of *HXT2*, *BTN2*, *IMD2* and *ECI1* from library plasmids led to a decrease in octanoic acid titers after 46 h of fermentation whereas individual knockouts did not have any effect on growth or octanoic acid titers (Fig.   4). Interestingly, growth and octanoic acid production were also delayed as a result of *HXT2*, *BTN2* and *IMD2* overexpressions (Fig. 3). In our RNA-Seq data, *HXT2* was found significantly downregulated at all sampling times. In addition, *HXT2* expression has also been found downregulated with externally supplied octanoic acid (Liu *et al*. 2013). As the downregulation was observable at all three sampling times, it seems to be rather independent of octanoic acid production. The expression of *BTN2*, similarly to *HXT2*, was downregulated at all sampling times in RNA-Seq (log_2_FC > 1, FDR < 0.01; except at T2: log_2_FC = 0.7) and its additional plasmid-based expression led to lower octanoic acid titers. *BTN2* encodes a v-SNARE protein involved in intracellular protein transport and regulation of pH (Chattopadhyay *et al*. 2000; Chattopadhyay, Roberts and Pearce 2003). In another study, *Δbtn2* strains showed an enhanced activity of vacuolar H^+^-ATPase as well as diminished growth and decreased buffering capacity under sorbic acid supply, affecting intracellular pH homeostasis (Chattopadhyay *et al*. 2000). This contradicts the downregulation of *BTN2* expression at all sampling times in the octanoic acid producer, as the presence of octanoic acid might create more acidic conditions which the cell has to counteract. Also, our *Δbtn2* strain did not show any difference in octanoic acid titers. The reason for the decrease in octanoic acid titers following plasmid-based *BTN2* expression is also unclear. These partly contradictory results could also arise from differences in protein turnover or possible post-translational modifications. The expression of *IMD2* was significantly upregulated in our RNA-Seq data at all three sampling times (log_2_FC > 1, FDR < 0.01; except at T1 log_2_FC = 0.65) and additional plasmid-based expression led to a 13% decrease in octanoic acid titers but did not influence growth. *IMD2* encodes an inosine monophosphate dehydrogenase, an enzyme involved in GTP biosynthesis. For *ECI1*, we observed a significant upregulation of expression at T3 (log_2_FC = 1.4) in our data as well as under octanoic acid supply in a non-producer strain in previously published data (Liu *et al*. 2013). *ECI1* encodes a peroxisomal isomerase and is essential for β-oxidation of unsaturated fatty acids (Geisbrecht *et al*. 1998). We observed that *ECI1* plasmid-based expression resulted in less octanoic acid whereas its knockout (LBY55) showed similar titers as the reference strain. We suspect that upregulation of *ECI1* leads to increased β-oxidation of unsaturated fatty acids, thereby possibly covering the cell's need for acetyl-CoA. Why this also leads to lower octanoic acid titers, remains obscure, as Eci1p has not been described to be involved in medium-chain fatty acid degradation. The exact effect of all four enzymes on octanoic acid biosynthesis remains to be investigated.

We found that most genes identified to be differentially expressed at more than one sampling time, did not have a significant effect on octanoic acid titers when overexpressed. This suggests that the upregulation of these genes in the octanoic acid producer had effects not directly related to the octanoic acid titers. One possibility is that the observed gene expression changes help to mitigate effects on precursor availability or for instance toxic effects of the acid.

The library plasmid containing two genes of interest, namely *RPL40B* and *PTR2*, led to a strong increase in octanoic acid titer after 46 h of fermentation. Further analysis showed that plasmid-based expression of *RPL40B* was responsible for the improvement. Interestingly, secreted C18:0 fatty acid titers were halved in this strain. As *RPL40B* expression was downregulated at T3 in the transcriptomics analysis (log_2_FC = 0.45), we suspect that this decrease reduces toxicity by lowering octanoic acid synthesis. In addition, a decrease in *RPL40B* expression may help maintain long-chain fatty acid synthesis at sufficient levels. Consequently, this would increase the strain's fitness. The genomic integration of a second copy of *RPL40B* under the control of the strong *pPGK1* promoter did not increase octanoic acid titers significantly and had no effect on C18 fatty acid titers. It seems that genomic expression of a second gene copy was not strong enough to exhibit the effects observed with expression from a multi-copy plasmid. Intriguingly, *RPL40B* expression from a multi-copy plasmid in non-producer CEN.PK2–1C, resulted in reduced growth. It is possible that this negative effect on growth is only observable in CEN.PK2–1C because LBY38 is already growth inhibited by inherent octanoic acid production. The decreased growth resulting from *RPL40B* overexpression in CEN.PK2–1C could also be attributed to lowered C18 fatty acid production by the native *FAS* complex and thereby impaired robustness. Rpl40b is a subunit of the ribosome (Fernández-Pevida *et al*. 2012) but to our knowledge has never been reported to play a direct role in fatty acid biosynthesis. Future work could investigate the global effect of increased Rpl40b expression via ribosome profiling (‘RiboSeq’) (Weinberg *et al*. 2016), proteome analyses and ribosome assembly studies. Another interesting approach could be to study knockout or knockdown mutants of *RPL40B* or create mutants of other ribosomal subunit-encoding genes such as *RPL40A*.

In the aforementioned published transcriptomic data sets (Legras *et al*. 2010; Liu *et al*. 2013), which were generated by adding octanoic acid externally to *S. cerevisiae* wild type strains, *RPL40B* had not been found to be differentially regulated. Overall, there was little overlap between our RNA-Seq data set and the two other data sets. On the one hand, the diverging results could be attributed to the difference in experimental conditions, such as strain background, media and pH. More importantly, differential expression seems to be highly growth phase-dependent and to differ for internally produced and externally supplied octanoic acid. This underlines the importance of collecting transcriptomic data not just for externally added compounds, but for actual producer strains. Our comprehensive transcriptomics data set facilitated the detection of an unreported genetic target by enabling an in-depth view into yeast physiology during octanoic acid production. We were able to identify the new target *RPL40B*, overexpression of which led to 40% more octanoic acid.

## MATERIALS AND METHODS

### Strains and plasmid construction

Yeast strains and plasmids used throughout this study are listed in Table S1 (Supporting Information). The genes *DAN1* and *NOP56* for overexpression were amplified from SHY34 (Wernig, Boles and Oreb 2019) genomic DNA with primers containing the respective overhangs for cloning via Golden Gate (Lee *et al*. 2015; oligonucleotides are listed in Table S2, Supporting Information). The *PDR12-tPDR12* fragment as well as promoter *pTDH3* were amplified from CEN.PK113–11C genomic DNA with primers containing the respective overhangs for cloning via homologous recombination into pRS42H/*Eco*RV. The library plasmid containing *RPL40B* and *PTR2* (A10–C10 from (Jones *et al*. 2008)) was split into two plasmids as follows: Each gene was amplified with its endogenous up- and downstream regions from the original plasmid with primers containing overhangs for the pGP564 backbone for homologous recombination in yeast (CEN.PK2–1C). pGP564 was digested with *Bam*HI/*Pst*I prior to use. Genomic knockout of *HXT2* was performed via CRISPR/Cas9 as described previously (Generoso *et al*. 2016). The CRISPR/Cas9 plasmid for *hxt2* knockout was assembled via Golden Gate—SiHV138 was used and the GFP-dropout region was replaced by pre-assembled, double stranded gRNA oligonucleotides. For *BTN2*, *IMD2* and *ECI1* knockout via CRISPR/Cas9 was not possible and ORFs were knocked out with the Cre-*loxP* recombinase system with the hphNT1 marker as described in (Güldener *et al*. 1996). For this purpose, the resistance cassette was amplified from plasmid pUG6H with primers containing overhangs for the up- and downstream regions, respectively, of the targeted ORF. For genomic integration of a second copy of *RPL40B*, the promoter *pPGK1* was amplified via PCR from pYTK011 and *RPL40B* was amplified via PCR from the original plasmid including the downstream part. Used primers contained suitable overhangs for the parts or *PYK2* up- or downstream regions, respectively. Integration was achieved via CRISPR/Cas9 plasmid LBV103 (assembled via homologous recombination into pRCC-K backbone) into the *PYK2* locus, thereby removing the entire *PYK2* ORF. Yeast transformations were performed according to Gietz and Schiestl (Gietz and Schiestl 2007) or for SHY34 via an adapted procedure (Gajewski *et al*. 2017). Cells were streaked out on selective YPD (1% yeast extract, 2% peptone, both by BD, Franklin Lakes, USA and Difco Laboratories, Franklin Lakes, USA; 2% D-glucose, Roth, Germany) containing hygromycin (200 µg/mL) to select for *hphNT1*, G-418 (200 µg/mL) to select for *kanMX* or on selective SCD medium (Bruder *et al*. 2016) lacking leucine (SCD^−leu^) to select for *LEU2*. Electrocompetent *E. coli* DH10β (Gibco BRL, Gaithersburg, MD) was used for subcloning according to standard procedures, and transformants were selected on lysogeny broth (LB) agar plates (Sambrook, Fritsch and Maniatis 1989) supplemented with carbenicillin (100 µg/mL), chloramphenicol (20 µg/mL) or kanamycin (50 µg/mL).

### Cultures for RNA-seq analysis

For pre-cultures, several colonies of SHY34 with plasmid *fusFAS^wt^* or *fusFAS^RK^*, respectively, were inoculated in 20 mL YPD with 100 mM potassium phosphate buffer adjusted to pH 6.5 and shaken (180 r.p.m.) at 30°C overnight. The main cultures were inoculated in triplicates to an OD_600_ of 0.1 in 50 mL buffered YPD medium in 300 mL flasks on a cell growth quantifier (Aquila Biolabs GmbH, Germany; Bruder *et al*. (2016)). Samples were taken for fatty acid quantification as described below. For RNA extraction, ∼10 OD units of cells/sample were pelleted in a pre-cooled falcon at 4°C (3000 rcf) for 3 min. Falcons were then immediately dropped into liquid nitrogen for a few minutes and stored at −80°C until further processing.

### RNA extraction, processing and sequencing

RNA extractions were performed on samples that were mechanically lysed with 0.5 mm acid washed beads using an MP-Biomedicals FastPrep-24 for three 1-minute cycles. Further extraction was performed using an RNeasy® Kit from Qiagen, Hilden, Germany. Libraries were prepared using the TrueSeq mRNA Stranded HT kit. Sequencing was carried out using an Illumina NextSeq 500 High Output Kit v2 (75 cycles), with a minimum of 10 million single-end reads per replicate. The Novo Nordisk Foundation Centre for Biosustainability (Technical University of Denmark) performed the RNA sequencing and library preparation. RNA-Seq data sets can be found using SRA accession PRJNA575618. RNA-Seq read mapping was performed after analysis in FASTQC. RNA-Seq mapping for differential expression was mapped with STAR (Dobin *et al*. 2013) and reads were assigned with featureCounts (Liao, Smyth and Shi 2014). Differential expression results can be found in Supplementary File 1.

### Cultures for fatty acid production

*Saccharomyces cerevisiae* strains were grown as previously described (Gajewski *et al*. 2017) with minor adjustments. For pre-cultures, several colonies of a strain were inoculated in 20 mL YPD with 100 mM potassium phosphate buffer adjusted to pH 6.5 and antibiotic (200 µg/mL hygromycin), if applicable, or in similarly buffered SCD medium lacking leucine (SCD^−leu^). After shaking (180 r.p.m.) at 30°C overnight, the main culture was inoculated to an OD_600_ of 0.1 in the respective medium and cultured in 300 mL shake flasks under the same conditions. For sampling, the cultures were harvested by centrifugation and 10 mL of the supernatant was used for fatty acid extraction.

### Fatty acid extraction and derivatization

Fatty acid extraction and derivatization were performed as described previously (Henritzi *et al*. 2018). Cells were separated from the medium (3500 rcf, 10 min), an internal standard (0.2 mg heptanoic acid) was added to 10 mL supernatant and mixed with 1 mL 1 M HCl and 2.5 mL methanol/chloroform solution (1:1). After vigorous shaking for 3 min, the mixture was centrifuged at 3000 rcf for 10 min and the chloroform layer was recovered and evaporated overnight. The methylation of fatty acids was performed as previously described (Ichihara and Fukubayashi 2010). Samples were dissolved in 200 µL toluene, mixed with 1.5 mL methanol and 300 µL 8.0% (w/v) HCl solution (diluted in methanol), vortexed and incubated at 100°C for 3 h to form fatty acid methyl esters (FAME). After cooling on ice for 10 min, 1 mL H_2_O and 1 mL hexane were added to the sample, followed by thorough shaking and the organic phase was transferred to a GC vial.

### GC-FID analysis of FAMEs

GC analyses were carried out on a Perkin Elmer Clarus 400 instrument (Perkin Elmer, Germany) equipped with an Elite FFAP capillary column (30 m × 0.25 mm, film thickness: 0.25 µm; PerkinElmer, Germany) and a flame ionization detector (Perkin Elmer, Germany) as described previously (Henritzi *et al*. 2018).

### Toxicity test

CEN.PK2–1C was transformed with plasmid pGP564 or *RPL40B*, respectively, and plated on SCD^−leu^. Pre-cultures were inoculated in triplicates in buffered SCD^−leu^ and grown over night at 30°C (180 r.p.m.). For main cultures, strains were inoculated to an OD_600_ of 0.2 and incubated for 4 h under the same condition (30°C, 180 r.p.m.). The cultures were then diluted in fresh media to an OD_600_ of 0.05 and transferred into a 96-well plate (clear with flat bottom) with 50 µL per well. A dilution series was made with octanoic acid diluted in the same media, to reach final concentrations in the wells of 0–300 mg/L. Of these dilutions, 200 µL were added per well. The three biological replicates per strain were inoculated in technical triplicates in the well plates. The starting absorbance was measured in a platereader (CLARIOstar®, BMG Labtech, Ortenberg, Germany) and plates were incubated for 20 h at 30°C without shaking before absorbance was measured again. From final absorbance values, a blank value (media without strain) was subtracted.

### Software

RNA-Seq read mapping and data analyses were performed with R packages limma, edgeR and piano. Data tables were stored in Microsoft Excel 2016. Fermentation graphs were made using the software Prism 9 (GraphPad, USA). Geneious Prime 2020.2 software was used for assembly planning.

### Data availability

RNA-Seq data sets of data generated in this study can be found using SRA accession PRJNA575618.

## ABBREVIATIONS

FAS: fatty acid synthase; FDR: false discovery rate; GC: gas chromatography; log_2_FC: log_2_ fold change; MPT: malonyl-palmitoyl transferase; OD_600_: optical density at λ = 600 nm.

## Supplementary Material

foab011_Supplemental_Files

## References

[bib1] Baumann L , Wernig F, Born S et al. Engineering *Saccharomyces cerevisiae* for production of fatty acids and their derivatives, In The MycotaVol. II: Genetics and Biotechnology, 3rd edn(Benz J. P., Schipper K., Eds), pp 339–68.. Springer, Switzerland. 2020.

[bib2] Besada-Lombana PB , Fernandez-Moya R, Fenster J et al. Engineering *Saccharomyces cerevisiae* fatty acid composition for increased tolerance to octanoic acid. Biotechnol Bioeng. 2017;114:1531–8. 28294288 10.1002/bit.26288

[bib3] Bruder S , Reifenrath M, Thomik T et al. Parallelised online biomass monitoring in shake flasks enables efficient strain and carbon source dependent growth characterisation of *Saccharomyces cerevisiae*. Microb Cell Fact. 2016;15:1–15. 27455954 10.1186/s12934-016-0526-3 PMC4960845

[bib4] Chattopadhyay S , Muzaffar NE, Sherman F et al. The yeast model for batten disease: mutations in btn1, btn2, and hsp30 alter pH homeostasis. J Bacteriol. 2000;182:6418–23. 11053386 10.1128/jb.182.22.6418-6423.2000 PMC94788

[bib5] Chattopadhyay S , Roberts PM, Pearce DA. The yeast model for Batten disease: a role for Btn2p in the trafficking of the Golgi-associated vesicular targeting protein, Yif1p. Biochem Biophys Res Commun. 2003;302:534–8. 12615067 10.1016/s0006-291x(03)00209-2

[bib6] Dobin A , Davis CA, Schlesinger F et al. STAR: ultrafast universal RNA-seq aligner. Bioinformatics. 2013;29:15–21. 23104886 10.1093/bioinformatics/bts635 PMC3530905

[bib7] Fernández-Pevida A , Rodríguez-Galán O, Díaz-Quintana A et al. Yeast ribosomal protein L40 assembles late into precursor 60 S ribosomes and is required for their cytoplasmic maturation. J Biol Chem. 2012;287:38390–407. 22995916 10.1074/jbc.M112.400564 PMC3488107

[bib8] Gajewski J , Pavlovic R, Fischer M et al. Engineering fungal de novo fatty acid synthesis for short chain fatty acid production. Nat Commun. 2017;8:14650. 28281527 10.1038/ncomms14650 PMC5353594

[bib9] Geisbrecht BV , Zhu D, Schulz K et al. Molecular characterization of *Saccharomyces cerevisiae* Δ3,Δ2-enoyl-CoA isomerase. J Biol Chem. 1998;273:33184–91. 9837886 10.1074/jbc.273.50.33184

[bib10] Generoso WC , Gottardi M, Oreb M et al. Simplified CRISPR-Cas genome editing for *Saccharomyces cerevisiae*. J Microbiol Methods. 2016;127:203–5. 27327211 10.1016/j.mimet.2016.06.020

[bib11] Gietz RD , Schiestl RH. High-efficiency yeast transformation using the LiAc/SS carrier DNA/PEG method. Nat Protoc. 2007;2:31–34. 17401334 10.1038/nprot.2007.13

[bib12] Güldener U , Heck S, Fiedler T et al. A new efficient gene disruption cassette for repeated use in budding yeast. Nucleic Acids Res. 1996;24:2519–24. 8692690 10.1093/nar/24.13.2519 PMC145975

[bib13] Henritzi S , Fischer M, Grininger M et al. An engineered fatty acid synthase combined with a carboxylic acid reductase enables de novo production of 1-octanol in *Saccharomyces cerevisiae*. Biotechnol Biofuels. 2018;11. 10.1186/s13068-018-1149-1 PMC5984327 29881455

[bib15] Huang CB , Alimova Y, Myers TM et al. Short- and medium-chain fatty acids exhibit antimicrobial activity for oral microorganisms. Arch Oral Biol. 2011;56:650–4. 21333271 10.1016/j.archoralbio.2011.01.011 PMC3119748

[bib14] Hu Y , Zhu Z, Nielsen J et al. Engineering *Saccharomyces cerevisiae* cells for production of fatty acid-derived biofuels and chemicals. Open Biol. 2019;9:190049. 31088249 10.1098/rsob.190049 PMC6544985

[bib16] Ichihara K , Fukubayashi Y. Preparation of fatty acid methyl esters for gas-liquid chromatography. J Lipid Res. 2010;51:635–40. 19759389 10.1194/jlr.D001065 PMC2817593

[bib17] Jarboe LR , Liu P, Royce LA. Engineering inhibitor tolerance for the production of biorenewable fuels and chemicals. Curr Opin Chem Eng. 2011;1:38–42.

[bib18] Jones GM , Stalker J, Humphray S et al. A systematic library for comprehensive overexpression screens in *Saccharomyces cerevisiae*. Nat Methods. 2008;5:239–41. 18246075 10.1038/nmeth.1181

[bib19] Klug L , Daum G. Yeast lipid metabolism at a glance. FEMS Yeast Res. 2014;14:369–88. 24520995 10.1111/1567-1364.12141

[bib20] Leber C , Choi JW, Polson B et al. Disrupted short chain specific β-oxidation and improved synthase expression increase synthesis of short chain fatty acids in *Saccharomyces cerevisiae*. Biotechnol Bioeng. 2016;113:895–900. 26388428 10.1002/bit.25839

[bib21] Lee ME , DeLoache WC, Cervantes B et al. A highly characterized yeast toolkit for modular, multipart assembly. ACS Synth Biol. 2015;4:975–86. 25871405 10.1021/sb500366v

[bib22] Legras JL , Erny C, Le Jeune C et al. Activation of two different resistance mechanisms in *Saccharomyces cerevisiae* upon exposure to octanoic and decanoic acids. Appl Environ Microbiol. 2010;76:7526–35. 20851956 10.1128/AEM.01280-10 PMC2976208

[bib23] Liao Y , Smyth GK, Shi W. FeatureCounts: an efficient general purpose program for assigning sequence reads to genomic features. Bioinformatics. 2014;30:923–30. 24227677 10.1093/bioinformatics/btt656

[bib24] Liu P , Chernyshov A, Najdi T et al. Membrane stress caused by octanoic acid in *Saccharomyces cerevisiae*. Appl Microbiol Biotechnol. 2013;97:3239–51. 23435986 10.1007/s00253-013-4773-5

[bib25] Rosenblatt J , Reitzel RA, Vargas-Cruz N et al. Caprylic and polygalacturonic acid combinations for eradication of microbial organisms embedded in biofilm. Front Microbiol. 2017;8:1999. 29093703 10.3389/fmicb.2017.01999 PMC5651231

[bib26] Sambrook J , Fritsch E, Maniatis T. Molecular Cloning: A Laboratory Manual. 2nd edn. Cold Spring Harb. Lab. Press. New York. (Evans G. A.Ed.) 1989.

[bib27] Sarria S , Kruyer NS, Peralta-Yahya P. Microbial synthesis of medium-chain chemicals from renewables. Nat Biotechnol. 2017;35:1158–66. 29220020 10.1038/nbt.4022

[bib28] Schmidt JH . Life cycle assessment of five vegetable oils. J Clean Prod. 2015;87:130–8.

[bib29] Viegas CA , Rosa MF, Sá-Correia I et al. Inhibition of yeast growth by octanoic and decanoic acids produced during ethanolic fermentation. Appl Environ Microbiol. 1989;55:21–28. 16347826 10.1128/aem.55.1.21-28.1989 PMC184048

[bib30] Viegas SC , Sá-Correia I, Novais JM. Synergistic inhibition of the growth of *Saccharomyces bayanus* by ethanol and octanoic or decanoic acids. Biotechnol Lett. 1985;7:611–4.

[bib31] Weinberg DE , Shah P, Eichhorn SW et al. Improved ribosome-footprint and mRNA measurements provide insights into dynamics and regulation of yeast translation. Cell Rep. 2016;14:1787–99. 26876183 10.1016/j.celrep.2016.01.043 PMC4767672

[bib32] Wernig F , Boles E, Oreb M. De novo biosynthesis of 8-hydroxyoctanoic acid via a medium-chain length specific fatty acid synthase and cytochrome P450 in *Saccharomyces cerevisiae*. Metab Eng Commun. 2019;e00111. 31867212 10.1016/j.mec.2019.e00111 PMC6906673

[bib33] Wernig F , Born S, Boles E et al. Fusing α and β subunits of the fungal fatty acid synthase leads to improved production of fatty acids. Sci Rep. 2020;10:9780. 32555375 10.1038/s41598-020-66629-y PMC7300031

[bib34] Yan Q , Pfleger BF. Revisiting metabolic engineering strategies for microbial synthesis of oleochemicals. Metab Eng. 2020;58:35–46. 31022535 10.1016/j.ymben.2019.04.009

