# Supplementary material for: Transcriptomic response of Saccharomyces cerevisiae to octanoic acid production
Source: FEMS Yeast Res. 2021 Feb 18;21(2):foab011. doi: 10.1093/femsyr/foab011 (PMC7972946; doi:10.1093/femsyr/foab011)
Supplement: foab011_Supplemental_Files [file foab011_supplemental_files.zip › Supplementary_Figures_and_Tables-Transcriptomic_response_to_octanoic_acid_production-1.pdf]

Transcriptomic response of *Saccharomyces cerevisiae* to octanoic acid production

Leonie Baumann,[1] Tyler Doughty,[2] Verena Siewers,[2] Jens Nielsen,[2,3] Eckhard Boles[1] and Mislav Oreb*[1]

[1] Institute of Molecular Biosciences, Faculty of Biological Sciences, Goethe University Frankfurt, Max-von-Laue Straße 9, 60438 Frankfurt am Main, Germany

[2] Department of Biology and Biological Engineering, Chalmers University of Technology, SE-41296 Gothenburg, Sweden

[3] Novo Nordisk Foundation Center for Biosustainability, Technical University of Denmark, 2800 Kongens Lyngby, Denmark

SUPPORTING INFORMATION

Table S1. Yeast strains and plasmids used in this study.

Strain	Characteristics	Reference
CEN.PK2-1C	*MATa*; *MAL2-8c*; *SUC2*; *ura3-52*; *his3Δ1; leu2-3_112*; *trp1-289*	Euroscarf, Frankfurt am Main, Germany
RPY21	*MATα*; *ura3Δ0*; *his3Δ0*; *leu2Δ0*; *TRP1*; *lys2Δ0*; *MET15*; Δ*FAS1::kanMX4*; Δ*FAS2::kanMX4; Δfaa2*; transformed with plasmids pRS315-$FAS1^{R1834K}$ and pRS313-*FAS2*	[1]
SHY34	*MATα*; *ura3Δ0*; *his3Δ0*; *leu2Δ0*; *TRP1*; *lys2Δ0*; *MET15*; *Δfas1 Δfas2 Δfaa2*	[2]
LBY38	CEN.PK2-1c Δ*pFAS1*-1-300::*pHXT7*-1-392 Δ*pFAS2*-1-200::*pHXT7*-1-392, $FAS1^{R1834K}$, *Δfaa2*	Boles laboratory
LBY49	LBY38 Δ*hxt2*	This study
LBY53	LBY38 Δ*btn2*	This study
LBY55	LBY38 Δ*eci1*	This study
LBY61	LBY38 Δ*pyk2*::*pPGK1-RPL40B-tRPL40B*	This study
**Plasmid**	**Characteristics**	**Reference**
**Plasmids used for fermentations**		
$fusFAS^{wt}$	pRS313-*pFAS-fusFAS12*-Wt	[3]
$fusFAS^{RK}$	pRS313-*pFAS-fusFAS12*-RK	[3]
pRS42H	2µ, HPH^R, *AmpR*, multiple cloning site including *Eco*RV	[4]
LBV16 (*PDR12*)	pRS42H with *pTDH3-PDR12-tPDR12* integrated in *Eco*RV site	This study
pGP564	2µ, *LEU2*, *KanR*	[5]
Library plasmids (containing genes): A10-F1 (*FAS1*), A16-E2 (*FAS2*), A15-A7 (*HIS3*/*YOR203W*), A15-D6 (*YOR186W*), A9-H11 (*MCH2*), A8-G4 (*IMD2*), A16-D6 (*EEB1*), A12-F8 (*HXT2*), A1-E10 (*MNC1*), A10-C10 (*PTR2*), A7-E2 (*BTN2*), A1-G6 (*URA7*), A5-A2 (*UTR2*), A3-B11 (*YDR133C*), A12-B2 (*INA1*), A14-D9 (*IZH4*) , A1-A8 (*PDR3*), A8-A2 (*SPS100*), A13-E10 (*SPS19*), A11-C10 (*ECI1*), A15-B2 (*RSB1*), A1-C1 (*GDH3*), A5-E11 (*HSP12*), A10-C10 (*RPL40B/PTR2*)	pGP564, yeast genomic DNA fragments of ~ 10 kb size	[5]
LBV100 (*PTR2*)	pGP564; 2µ, *LEU2*, *KanR*, *pPTR2-PTR2-*tPTR2	This study
LBV101 (*RPL40B*)	pGP564; 2µ, *LEU2*, *KanR*, *pRPL40B-*RPL40B-*tRPL40B*	This study
SiHV010	2µ, HPH^R, *KanR*	[6]
LBV71	SiHV010-*pYEF3-DAN1-tSSA1*	This study
LBV72	SiHV010-*pYEF3-NOP56-tSSA1*	This study
**Plasmids used for CRISPR/Cas9**		
pRCC-K	2µ, *kanMX*, *AmpR*, *pROX3-*$Cas9^{opt}$*-tCYC1*, *pSNR52-gRNA*	[7]

SiHV138	2μ, *kanMX*, *AmpR*, *pROX3-Cas9^{opt}-tCYC1*, *pSNR52-gRNA, GFP-dropout* (modified from pRCC-K)	7
LBV52	SiHV138 with *gRNA* (TGGGTTAACCTTAGGACGTC) for *HXT2* locus	This study
LBV103	pRCC-K with gRNA for *PYK2* (ATGTCTTTGGCGGACAAGGG) locus	This study
**Plasmid used for Golden Gate assembly**		
pYTK001	Golden Gate entry vector, *CamR*	6
**Plasmids Cre-*loxP* recombinase system**		
pUG6H	contains *loxP-HPH^R-loxP* for knockout in yeast	8

Table S2. Oligonucleotides used in this study.

Primer	Sequence 5'-3'	Application
**Cloning of LBV71, LBV72**		
LBP321	CGTCTCGTCGGTCTCATATGTCTAGAATTAGTATATTAGCTGTCG	Amplification of *DAN1* from SHY34 genomic DNA with overhangs for Golden Gate cloning
LPB322	CGTCTCAGGTCGGTCTCAGGATCTATAACAATAGAGCGGCGGC	
LBP323	CGTCTCGTCGGTCTCATATGGCTCCTATTGAATACCTAC	Amplification of *NOP56* from SHY34 genomic DNA with overhangs for Golden Gate cloning
LBP324	CGTCTCAGGTCGGTCTCAGGATTTAATCCTTACTTTTCTTCTTTTTATCCTTC	
SiHSeq001	TCCTGGCCTTTTGCTGG	Sequencing of *DAN1* and NOP56 in pYTK001 backbone
SiHSeq002	GGACTCCTGTTGATAGATC	
**Cloning of LBV100, LBV101**		
LBP406	CTCCACCGCGGTGGCGGCCGCTCTAGAACTAGTGTGCTATGTATTCTTAGTGTACTGC	Amplification of *PTR2* including up- and downstream regions from A10-C10 of library[5]
LBP407	GTCGACGGTATCGATAAGCTTGATATCGAATTCTCGATTTATTACGATCTCCACAAATC	
LBP408	GCTCCACCGCGGTGGCGGCCGCTCTAGAACTAGTGTGCGTTTAATTAACTTACTGTC	Amplification of *RPL40B* including up- and downstream regions from A10-C10 of library[5]
LBP409	CGAGGTCGACGGTATCGATAAGCTTGATATCGAATTCTATCCTAAAACGTGGCGTTA	
LBP237	GGAATTGTGAGCGGATAAC	Sequencing of LBV100, LBV101
LBP238	ACTATAGGGCGAATTGGG	
**Cloning of LBV16**		
LBP72	CCTCGAGGTCGACGGTATCGATAAGCTTGATATCACAGTTTATTCCTGGCATCCAC	Amplification of *pPGK1* from CEN.PK113-11C with overhangs to *PDR12* and pRS42H, respectively
LBP73	GTCTTTCTCAATATGTTCGTCAGTCGAAGACATTTTGTTTGTTTATGTGTGTTTATTCG	
LBP74	CGAATAAACACACATAAACAAACAAAATGTCTTCGACTGACGAACATATTGAGAAAGAC	Amplification of *PDR12-tPDR12* from CEN.PK113-11C with overhangs to pRS42H and *pTDH3*, respectively
LBP75	GATCCCCCGGGCTGCAGGAATTCGATGATATCTCCTTTTGAAGGTGATAGGGATATAATG	
LBP85	GGCGATTAAGTTGGGTAACG	Sequencing of LBV16
RPP056	CACACAGGAAACAGCTATGAC	

**Knockout of *BTN2*, *IMD2* and *ECI1* via Cre-*loxP* recombinase system**		
LBP325	CAACCAAAAGAAAATAACTAATAGACCCCATTACAATATAGAATTCGTACGCTGCAGGTCGAC	Amplification of HPH^R cassette with overhangs for *BTN2* up/downstream regions
LBP326	CGTAAAAATGAAAGATGGGGAGTATGTATTATCACCCAGCATAGGCCACTAGTGGATCTG	
LBP329	ATAATCAGTGCAATTAATAACTCCACAAGTAGCAAAAGCATTCGTACGCTGCAGGTCGAC	Amplification of HPH^R cassette with overhangs for *IMD2* up/downstream regions
LBP330	CATCAGTAATACTGTATTGATGATGCCATTTTAACATGCATAGGCCACTAGTGGATCTG	
LBP333	GGCACAAATTGCTCGCACAGTAAAGGAAGGAAGAACAATTTCGTACGCTGCAGGTCGAC	Amplification of HPH^R cassette with overhangs for *ECI1* up/downstream regions
LBP334	ATTGTGTGTGCGTTTTGTTTCACTGAGAAAGCGGACGGCATAGGCCACTAGTGGATCTG	
LBP309	GTCATGTAGCACTATTTCAGCC	Sequencing of *BTN2* locus
LBP310	GAATCACTTGCCATTCCACC	
LBP327	ACCATTACAGACCAACTTAC	
LBP328	AACTTCCTCATCCTCAAC	
LBP311	AGAGATGATGCAAGAGTAG	Sequencing of *IMD2* locus
LBP312	TCACCACCAAAATCACC	
LBP331	ACCAAGAGCCTACCAAGAC	
LBP332	CCAACATACCACCCATCATAAC	
LBP335	TCACTTAATGAACCCTGAC	Sequencing of *ECI1* locus
LBP336	CTCTCCATCTACCCAATAC	
LBP337	TCAAATAAACCTGCTGCC	
LBP338	ACGCAACTAAATGAGCAC	
**Knockout of *HXT2***		
LBP317	GATCTGGGTTAACCTTAGGACGTC	Amplification of gRNA for *HXT2* locus
LBP318	AAACGACGTCCTAAGGTTAACCCA	
LBP320	GATTATAAGAACAACAAATTAAATTACAAAAAGACTTATAAAGCAACATAGAGATTATACTTAAACTAGCACTGATTTTTTTAAGGCTAATGGCTACTAA	Donor for *hxt2* knockout
MGP126	GGGAAACGCCTGGTATC	Sequencing of gRNAs
LBP313	TCCCTCTCCACTCTTTCTC	Sequencing of *HXT2* locus
LBP314	ATCAGCCACAATACGCC	
**Integration of *pPGK1-RPL40B* into *PYK2***		
LBP106	ATGTCTTTGGCGGACAAGGGGTTTTAGAGCTAGAAATAGCAAGTTAAAATAAGG	Amplification of gRNA for *PYK2* locus
LBP107	CCCTTGTCCGCCAAAGACATGATCATTTATCTTTCACTGCGGAG	
LBP430	CATATTATTTCGAACATAGTATTCTGTATTCGCCGTAACATACATTTGCATGATCTTGTTTTATATTTGTTGTAAAAAG	Amplification of *pPGK1* from pYTK001 with overhangs to *pyk2*-downstream and *RPL40B*, respectively
LBP431	CTGACACAATGGACAATTAAATAAAATTAAGTAAAAAAAATAAGGACTTTAATTTTTAAACGGTGAGTAAGGAAAG	
LBP428	CTATATTTTACTTTCATCCTCTACGTCCATTGTAAGATTACAACAAAAGCACTATCGTGCGTTTAATTAACTTACTGTC	Amplification of *RPL40B* with downstream region from A10-C10 library[5]
LBP429	CAGATCATCAAGGAAGTAATTATCTACTTTTTACAACAAATATAAAACAAGATCATGCAAATGTATGTTACGG	
LBP118	CAGAGCGGTGAAACGCAAC	Sequencing of △*pyk2*::*pPGK1-RPL40B-tRPL40B*
LBP119	CGCAGTTTGCGAACATTACCTG	
VSP159	CGTGTGACAACAACAGCC	

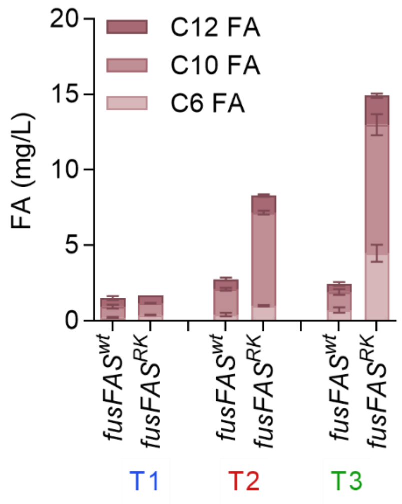

Figure S1: Characterization of C6, C10 and C12 fatty acid production. Strains SHY34+*fusFAS*^{wt} (reference strain) and SHY34+*fusFAS*^{RK} (octanoic acid producer) were cultured for 46 h in buffered YPD medium. Sampling times are indicated with arrows (T1 = 14 h; T2 = 22 h; T3 = 46 h). Fatty acids (FA) were extracted and quantified via GC. *n* = 3, error bars = ± standard deviation.

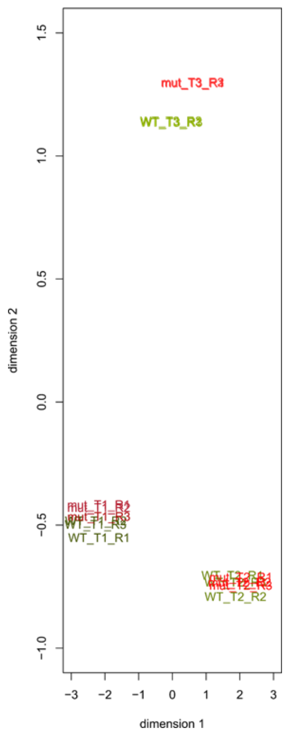

Figure S2: Principal component analysis (PCA) of RNASeq data sets. The RNASeq data of all three replicates (R1-R3) of the three sampling times (T1 = 14 h; T2 = 22 h; T3 = 46 h) of the reference strain (WT) and the octanoic acid producer strain (mut), respectively, were compared in PCA suggesting similar global expression.

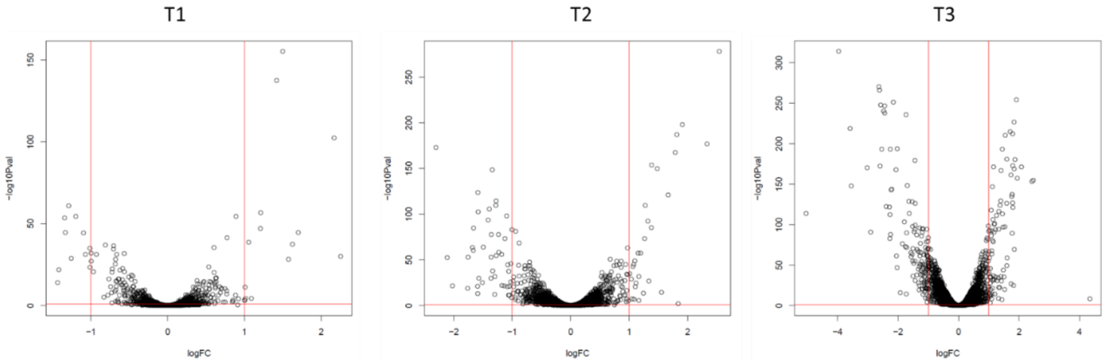

Figure S3: Differential gene expression analysis. Volcano plots show the $\log_2$FC of gene expression at the three sampling times (T1 = 14 h; T2 = 22 h; T3 = 46 h) for SHY34+*fusFAS*^{RK} (octanoic acid producer) compared to SHY34+*fusFAS*^{WT} (reference strain).

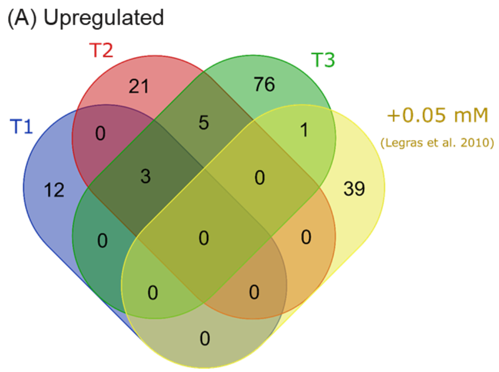

Overlaps with…	Gene
…T1	*PDR12*

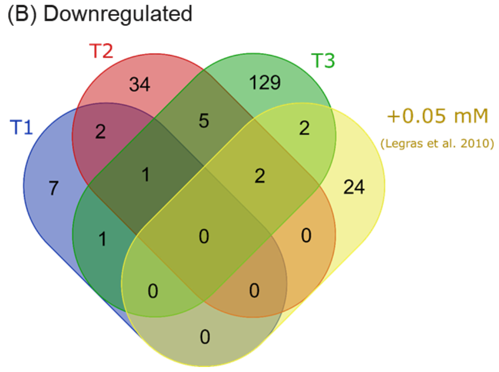

Overlaps with…	Gene
…T2, T3	*URA7*
	INA1
…T3	*ZRT1*
	MKC7

Figure S4: Comparison of transcriptome data of an octanoic acid producer with the response to externally supplied octanoic acid to a non-producer. Number of genes that are upregulated (A) or downregulated (B) in the two experiments. For RNA-Seq, an octanoic acid producer strain was analyzed in comparison to a non-producer strain ($\log_2$FC > 1) at different sampling times (T1 = 14 h; T2 = 22 h; T3 = 46 h). Microarray differential expression data of a non-producer strain supplied with 0.05 mM octanoic acid for 20 minutes in comparison to non-supplied control ($\log_2$FC ≥ 0.3); data taken from Legras *et al*. 2010.[9] Gene lists include genes that overlap between data from Legras *et al*. 2010 and at least one sampling time of the RNA-Seq experiment.

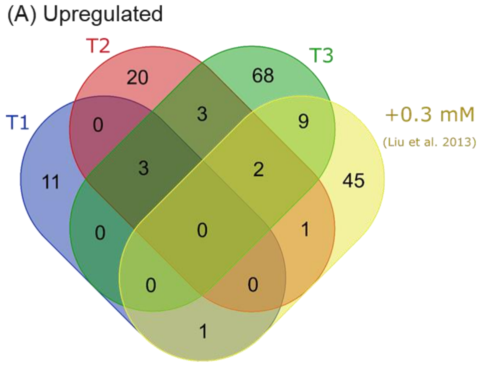

Overlaps with…	Gene
…T2, T3	*IMD2*
	EEB1
…T1	*PHO5*
…T2	*PDC6*
…T3	*PDR3*
	SPS100
	POX1
	SPS19
	ECI1
	PDR15
	RSB1
	PDR12
	GDH3

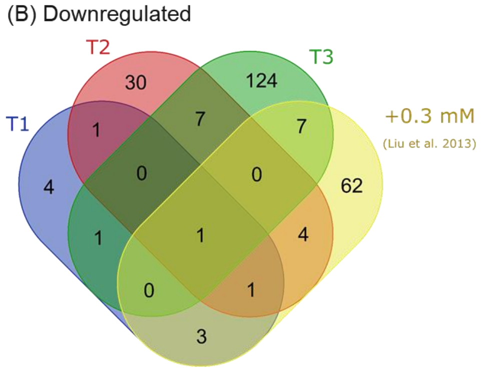

Overlaps with…	Gene
…T1, T2, T3	*HXT2*
…T1, T2	*PTR2*
…T1	*SPL2*
	PHO84
	MMP1
…T2	*AGP1*
	HXT4
	GAP1
	PUT4
…T3	*DBP2*
	SUR7
	HO
	ARO9
	RNH201
	HSP12
	ARO10

Figure S5: Comparison of transcriptome data of an octanoic acid producer with the response to externally supplied octanoic acid to a non-producer. Number of genes that are upregulated (A) or downregulated (B) in the two experiments. For RNA-Seq, an octanoic acid producer strain was analyzed in comparison to a non-producer strain ($log_2FC > 1$) at different sampling times (T1 = 14 h; T2 = 22 h; T3 = 46 h). Microarray differential expression data of a non-producer strain supplied with 0.3 mM octanoic acid in comparison to non-supplied control ($log_2FC > 1$) in mid-log growth; data taken from Liu *et al*. 2013.[10] Gene lists include genes that overlap between data from Liu *et al*. 2013 and at least one sampling time of the RNA-Seq experiment.

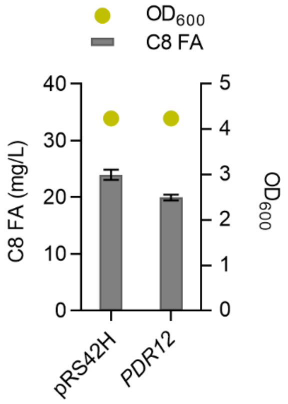

Figure S6: Effect of *PDR12* overexpression. The octanoic acid producer strain RPY21-*FAS1*^{RK}-*FAS2* was transformed with a multi-copy plasmid containing *PDR12* or the control vector pRS42H. Strains were cultured in buffered YPD^{hygro} medium and fatty acids were extracted after 72 h and quantified by GC measurement. *n* = 2, error bars = ± standard deviation.

REFERENCES

(1) Henritzi, S., Fischer, M., Grininger, M., Oreb, M., and Boles, E. (2018) An engineered fatty acid synthase combined with a carboxylic acid reductase enables de novo production of 1-octanol in *Saccharomyces cerevisiae*. *Biotechnol. Biofuels 11*.

(2) Wernig, F., Boles, E., and Oreb, M. (2019) *De novo* biosynthesis of 8-hydroxyoctanoic acid via a medium-chain length specific fatty acid synthase and cytochrome P450 in *Saccharomyces cerevisiae*. *Metab. Eng. Commun.* e00111.

(3) Wernig, F., Born, S., Boles, E., Grininger, M., and Oreb, M. (2020) Fusing α and β subunits of the fungal fatty acid synthase leads to improved production of fatty acids. *Sci. Rep. 10*, 9780.

(4) Taxis, C., and Knop, M. (2006) System of centromeric, episomal, and integrative vectors based on drug resistance markers for *Saccharomyces cerevisiae*. *Biotechniques 40*, 73–78.

(5) Jones, G. M., Stalker, J., Humphray, S., West, A., Cox, T., Rogers, J., Dunham, I., and Prelich, G. (2008) A systematic library for comprehensive overexpression screens in *Saccharomyces cerevisiae*. *Nat. Methods 5*, 239–241.

(6) Lee, M. E., DeLoache, W. C., Cervantes, B., and Dueber, J. E. (2015) A Highly Characterized Yeast Toolkit for Modular, Multipart Assembly. *ACS Synth. Biol. 4*, 975–986.

(7) Generoso, W. C., Gottardi, M., Oreb, M., and Boles, E. (2016) Simplified CRISPR-Cas genome editing for *Saccharomyces cerevisiae*. *J. Microbiol. Methods 127*, 203–205.

(8) Güldener, U., Heck, S., Fiedler, T., Beinhauer, J., and Hegemann, J. H. (1996) A new efficient gene disruption cassette for repeated use in budding yeast. *Nucleic Acids Res. 24*, 2519–2524.

(9) Legras, J. L., Erny, C., Le Jeune, C., Lollier, M., Adolphe, Y., Demuyter, C., Delobel, P., Blondin, B., and Karst, F. (2010) Activation of two different resistance mechanisms in *Saccharomyces cerevisiae* upon exposure to octanoic and decanoic acids. *Appl. Environ. Microbiol. 76*, 7526–7535.

(10) Liu, P., Chernyshov, A., Najdi, T., Fu, Y., Dickerson, J., Sandmeyer, S., and Jarboe, L. (2013) Membrane stress caused by octanoic acid in *Saccharomyces cerevisiae*. *Appl. Microbiol. Biotechnol. 97*, 3239–3251.
